# Past, present, and future of thermogenic fat research: A bibliometric analysis from 2000 to 2023

**DOI:** 10.1097/MD.0000000000049210

**Published:** 2026-06-12

**Authors:** Haiyan Xie, Lan Chen, Boyuan Long, Xudong Song, Mingsheng Jiang, Ziyi Song

**Affiliations:** a Guangxi Key Laboratory of Animal Breeding, Disease Control and Prevention, College of Animal Science and Technology, Guangxi University, Nanning, China.

**Keywords:** beige fat, bibliometric analysis, brown fat, obesity, thermogenic fat

## Abstract

**Background:**

Thermogenic fat plays a crucial role in regulating energy metabolism and improving obesity-related metabolic diseases. However, a systematic analysis of the research trends and hotspots in the field of thermogenic fat is lacking. This study aimed to fill this gap by employing bibliometric methods to analyze the global research landscape of thermogenic fat from 2000 to 2023.

**Methods:**

A systematic search was conducted in the Web of Science Core Collection to retrieve publications related to thermogenic fat. Various bibliometric tools (HistCite, CiteSpace, and VOSviewer) were used to analyze and visualize the data, including annual publication trends, geographical distribution, institutional and author contributions, citation analysis, and keyword analysis.

**Results:**

A comprehensive search yielded a total of 5246 English-language articles, revealing a significant upward trend in the number of publications focused on thermogenic fat over the past 24 years. The United States stands out as the most productive country, with Harvard University emerging as the leading research institution in this field. Bruce M. Spiegelman was identified as a pivotal figure in advancing thermogenic fat research. The journals that have published the highest number of articles in this field include Molecular Metabolism, Journal of Biological Chemistry, and Scientific Reports, with Cell Metabolism receiving the highest number of citations. The primary research keywords cluster into 9 distinct categories, with the most frequently occurring terms being “obesity,” “brown adipose tissue,” “thermogenesis,” “brown adipocytes,” “energy expenditure,” “browning,” “UCP1,” “beige fat,” and “type 2 diabetes.” Notably, white fat browning and beige fat have emerged as cutting-edge research trends.

**Conclusion:**

This investigation provides a comprehensive overview of the global research trends in thermogenic fat through bibliometric analysis. Enhanced insights into the historical development and evolutionary trajectory of this field offer a novel perspective on potential future research avenues. The findings of this study are poised to serve as an invaluable resource for researchers, thereby fostering continued advancement in the study of thermogenic fat.

## 1. Introduction

In mammals, adipose tissue is categorized into 3 distinct types: white adipose tissue (WAT), brown adipose tissue (BAT), and beige adipose tissue. Brown and beige adipose tissues are often referred to collectively as thermogenic fat. WAT primarily stores energy in the form of triglycerides, while BAT is rich in mitochondria and expresses high levels of uncoupling protein 1 (UCP1). BAT mediates thermogenesis in response to cold exposure or feeding, helping maintain body temperature and serving as a crucial organ for clearing triglycerides and controlling blood sugar levels. Beige adipose tissue is a unique type that is scarcely present under normal physiological conditions. However, when the body experiences chronic cold exposure or other stimuli, white adipocytes or their progenitor cells can undergo transdifferentiation or differentiation into brown-like adipocytes, known as beige adipocytes. This phenomenon is known as “white fat browning.”^[1,2]^ Recent research has shown that increasing the mass of thermogenic fat can enhance energy expenditure, reduce body fat content, and alleviate various obesity-related metabolic diseases, including type 2 diabetes mellitus, nonalcoholic fatty liver disease, cardiovascular disease, and even cancer.^[3,4]^ Consequently, the study of thermogenic fat has emerged as a significant research focus in the field of metabolic biology.

Bibliometrics is a quantitative method that utilizes mathematical and statistical techniques to analyze large volumes of literature data. It aims to elucidate the knowledge structure and emerging trends within a specific research topic or field. The earliest studies related to bibliometrics date back to 1950.^[5]^ Over time, bibliometrics has emerged as a recognized scientific discipline and an integral component of research evaluation methodologies.^[6,7]^ Bibliometric analysis techniques can be broadly categorized into performance analysis and science mapping. Performance analysis focuses on quantifying the contributions of research components (countries, institutions, authors, and topics), while science mapping emphasizes examining the relationships between these components.^[8–11]^ By studying the social and structural relationships among countries, institutions, authors, and topics within a field, as well as annual publication counts, citation patterns, and co-citation analyses, we can gain insights into the field’s knowledge dynamics and influence. However, to date, no studies have applied bibliometric analysis to thermogenic fat research. Therefore, this study aims to fill this gap by utilizing bibliometric methods and 3 visualization tools (HistCite, CiteSpace, and VOSviewer) to analyze the published literature in this field from 2000 to 2023. The objective is to qualitatively and quantitatively reveal the past, present, and future trends in thermogenic fat research. This analysis provides valuable guidance for researchers in this field.

## 2. Materials and methods

### 2.1. Data extraction

The Web of Science, recognized as the world’s largest and most comprehensive compilation of information resources, has been widely accepted by researchers as a high-quality digital literature resource database, particularly suitable for bibliometric analysis.^[12]^ To ensure the accuracy and precision of retrieved data, our study exclusively utilized the Science Citation Index Expanded database within the Web of Science Core Collection (WoSCC). We carefully selected publications and conducted our search on January 11, 2024, to minimize biases resulting from daily database updates.

**Figure 1.**
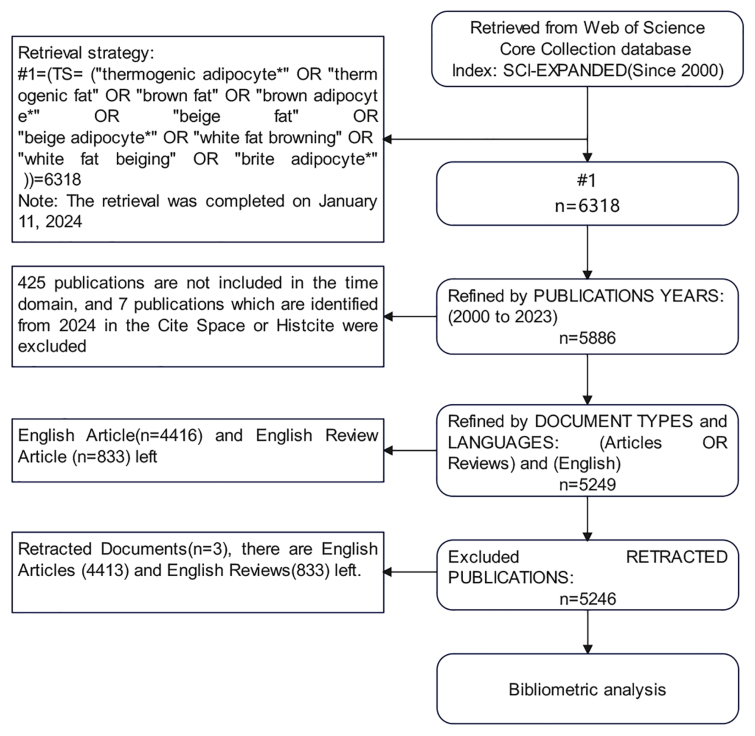
Flowchart of document selection and identification. SCIE = Science Citation Index Expanded.

We employed an advanced search strategy using topic searches, combining multiple synonyms for key terms and applying restrictions on publication year, document type, and language. The final search strategy was: TS= (“thermogenic adipocyte*” OR “thermogenic fat” OR “brown fat” OR “brown adipocyte*” OR “beige fat” OR “beige adipocyte*” OR “white fat browning” OR “white fat beiging” OR “brite adipocyte*”). The asterisk (*) indicates that the preceding noun should be included in both singular and plural forms in the search filter. We limited the publication years to 2000 to 2023, selected document types as “Article” or “Review Article,” and specified the language as English. All retrieved data were exported in the “full record and cited references” format, saved as txt files with the prefix “download_.” These files were used for subsequent analysis. After excluding 3 retracted articles (Table S1, Supplemental Digital Content 1) and 7 articles with incorrect publication years, we obtained a total of 5246 articles, including 4413 research articles and 833 review articles (Table S2, Supplemental Digital Content 2). The search strategy used in this study is illustrated in Figure 1.

### 2.2. Data analysis

HistCite (Clarivate Analytics) was utilized in this study to analyze publication counts, total global citation scores (TGCS), and total local citation scores (TLCS) for articles related to thermogenic fat. TGCS represents the number of citations in the WoSCC, while TLCS reflects citations within the current database, which can reveal the popularity of the article in this field and is usually less than TGCS.^[13]^ These metrics were examined for each publication year, active country, active institution, active author, and core journal.

CiteSpace, developed by Professor Chen Chaomei at Drexel University in 2004, is a Java application designed to analyze and visualize co-citation networks. It can detect and visualize emerging trends and fundamental changes in scientific disciplines over time.^[14]^ In this study, CiteSpace was used to analyze keyword burst detection maps and country betweenness centrality for thermogenic fat. The time slicing was set from January 2000 to December 2023, with each slice representing 2 years. Node types were set to “keyword,” “country,” and “institution,” and the selection criteria were set to the top 50 most cited or frequent items per slice. Modularity *Q* and average silhouette scores were used to evaluate the reliability of clustering, with *Q* > 0.3 and average silhouette >0.5 indicating sufficient cluster structure and convincing cluster results, respectively.^[15]^ Betweenness centrality, a metric to assess a node’s influence and importance within a network,^[16]^ was calculated to identify influential countries in the research field.

VOSviewer (CWTS, Leiden University) facilitates the analysis of relationships between publications by identifying the most influential works in a research area. Co-citation analysis, a method to understand the development of fundamental themes within a research field by analyzing relationships between cited publications,^[15]^ is enhanced by VOSviewer through integrated network visualization, spectral clustering, automatic cluster labeling, and text summarization. In this study, VOSviewer was employed to generate collaboration cluster maps among countries, institutions, and authors; co-citation clusters among journals; co-occurrence clusters among keywords; and timeline visualizations. In these visualizations, each institution or author is represented as a node, and collaborations are depicted as links between nodes. Node size correlates with collaboration strength, and line thickness represents the intensity of collaborative links, and node color indicates cluster membership.^[17]^

Microsoft Excel (Microsoft Corporation) was utilized to construct tables and bar charts, facilitating the visualization of pertinent data pertaining to countries, authors, institutions, and references.

## 3. Results

### 3.1. An overview of thermogenic fat-related publications

**Figure 2.**
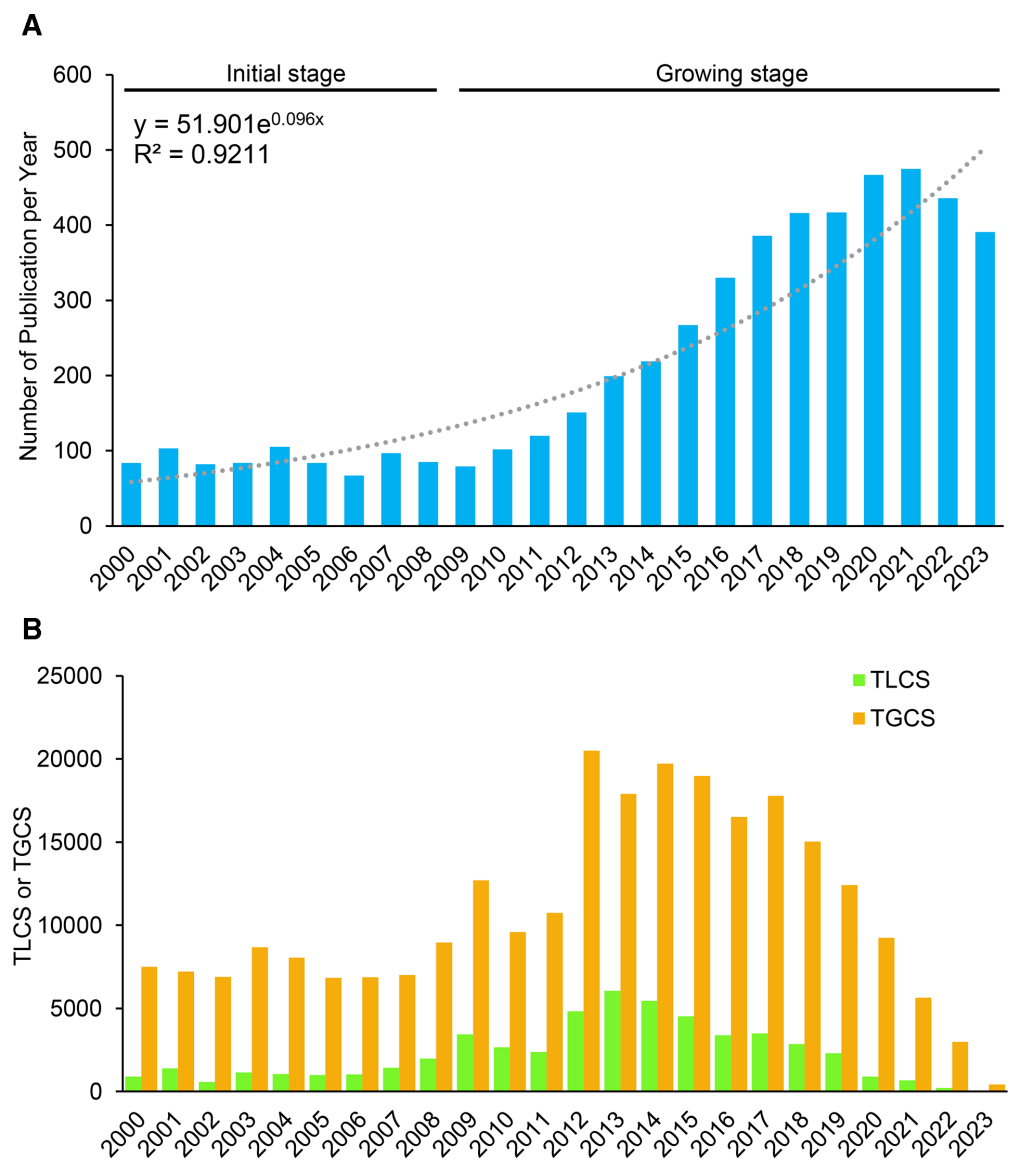
Publication outputs and citations on thermogenic adipocytes from 2000 to 2023. (A) Global annual production trends. Blue bars represent the number of publications related to thermogenic adipocytes per year, and the dotted line represents the trend-fitted curve. (B) Annual TGCS and TLCS of publications. TGCS = total global citation score, TLCS = total local citation score.

Over the past 24 years, a total of 5246 documents have been published in this field. There has been a notable upward trend in global publications in the field of thermogenic fat, increasing from 84 publications in 2000 to 391 publications in 2023 (Fig. 2A). To characterize the publication trend, exponential fitting was performed using the extracted data of the annual output over the past 24 years (2000–2023; Fig. 2A). The fitting equation of the exponential curve was *y* = 51.901e^0.096x^ (with a correlation coefficient of 0.9211). Based on the global annual publication trend, the period can be divided into 2 phases: an initial stage from 2000 to 2008 and a rapid growth stage from 2009 to 2023. From 2000 to 2008, the annual number of articles in this field exhibited fluctuating growth and remained relatively low, with a maximum of 105 articles, indicating slow progress and a lack of creative breakthroughs during this period. However, from 2009 to 2021, publications on thermogenic fat research entered a rapid development stage, with a total of 3628 articles and an average of approximately 279.08 articles per year, suggesting that significant breakthroughs were achieved in this field around 2008 to 2009. Although there was a slight decline from 2021 to 2023, the overall trend remained steady (Fig. 2A). Next, citation analysis showed that publications in this field have been cited a total of 258,192 times in the WoSCC, with an average citation rate of 49.22 times per article. Consistent with the annual publication trend, the TGCS and TLCS were relatively low during the initial stage but gradually increased from 2005 to 2012, peaking in 2012 with 20,498 citations, suggesting that this field has attracted increasing attention from researchers and has become a research hotspot (Fig. 2B).

### 3.2. Countries leading in thermogenic fat research

**Figure 3.**
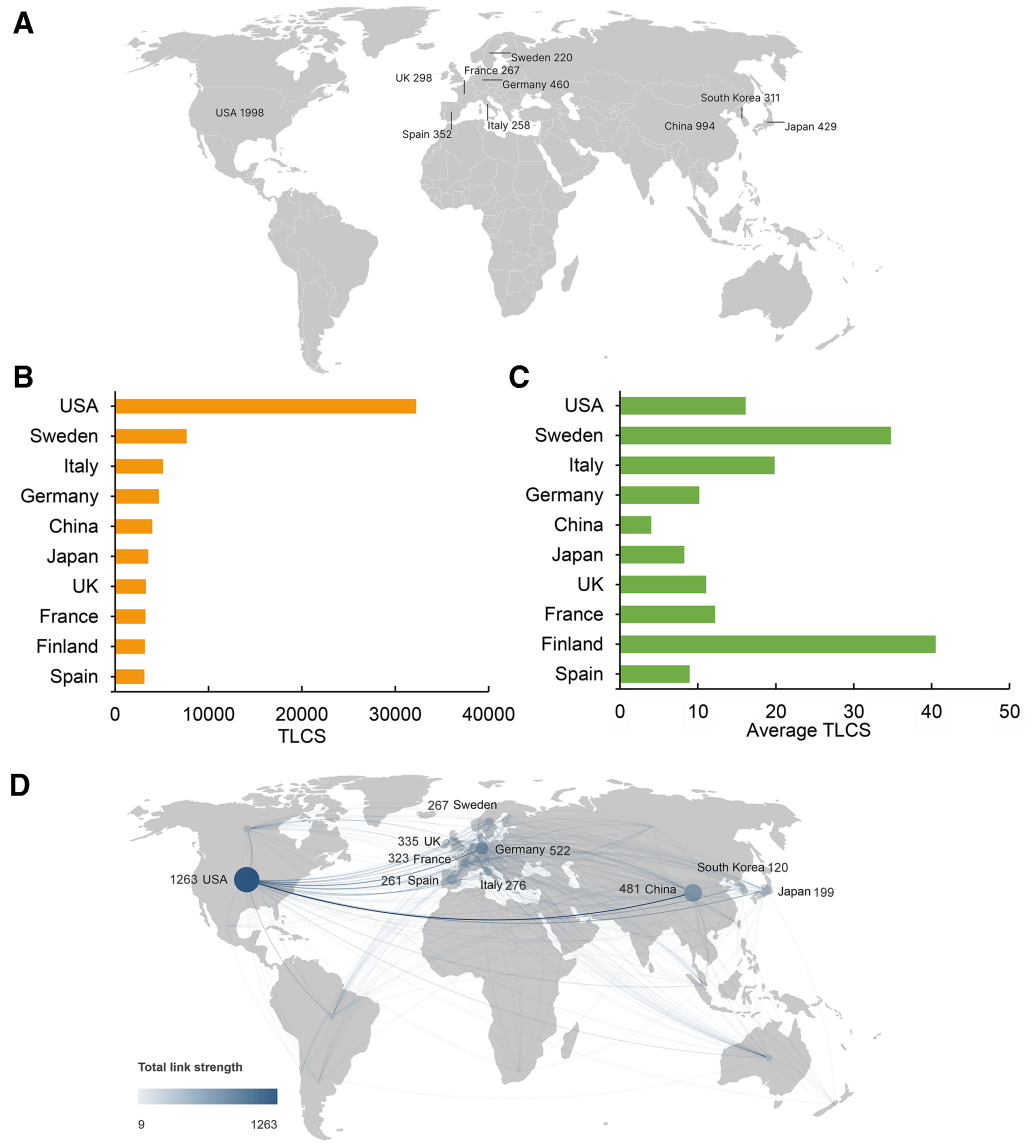
The top 10 productive countries concerning thermogenic adipocytes. (A) The top 10 productive countries concerning thermogenic adipocytes. (B, C) TLCS and average TLCS of the top 10 countries with the highest TLCS. (D) The international collaboration among different countries. Each country is represented as a node, and each line represents a co-authorship relationship. The node size is proportional to the collaboration link strength. TLCS = total local citation score.

In the past 24 years, a total of 82 countries and regions have published papers in the field of thermogenic fat. Among them, the United States was the primary contributor to thermogenic fat research, accounting for 38.09% of studies (1998 articles), followed by China (994 articles), Germany (460 articles), Japan (429 articles), and Spain (352 articles) (Fig. 3A, Table S3, Supplemental Digital Content 3). Consistently, studies from the United States had the highest number of TGCS citations (146,131), followed by China (25,163), Germany (24,528), Switzerland (20,778), and Italy (20,303; Table S3, Supplemental Digital Content 3). Compared with TGCS, the top 5 countries with the highest TLCS were the United States (32,243), Switzerland (7649), Italy (5118), Germany (4689), and China (3992; Fig. 3B). These results further demonstrated the leading role of the United States in thermogenic fat research.

Next, the co-authorship analysis of 46 countries that have published more than 10 publications revealed collaboration relationships among countries in the thermogenic fat field (Fig. 3D). The highest total link strength was observed in the United States (total link strength = 1263), followed by Germany (522), China (481), the United Kingdom (335), France (323), and Italy (276; Fig. 3D). Betweenness centrality is an indicator of a node’s influence on the network, where nodes with higher betweenness centrality have a greater impact on information transmission through the network. Higher centrality in the collaboration network corresponds to closer cooperation. Consistent with total link strength, the United States had the highest betweenness centrality in collaborations (Table 1), indicating its closer cooperation with other countries and further highlighting its central role in leading global thermogenic fat research.

**Table 1:** Top 10 countries with the highest TLCS.

Rank	Country	Publications	TLCS	TGCS	Average TLCS	Betweenness centrality
1	USA	1998	32,243	146,131	16.14	0.44
2	Sweden	220	7649	20,778	34.77	0.12
3	Italy	258	5118	20,303	19.84	0.08
4	Germany	460	4689	24,528	10.19	0.18
5	China	994	3992	25,163	4.02	0.09
6	Japan	429	3540	16,551	8.25	0.14
7	UK	298	3295	17,855	11.06	0.17
8	France	267	3256	16,625	12.19	0.11
9	Finland	79	3201	8122	40.52	0.01
10	Spain	352	3147	16,778	8.94	0.04

TGCS = total global citation score, TLCS = total local citation score.

### 3.3. Active institutes in thermogenic fat research

A total of 21,584 institutions contributed to publications on thermogenic fat. Among the top 10 productive institutions, there were 4 American institutions, ranking first, followed by 2 Chinese institutions. Specifically, Harvard University published the most articles (181 publications), followed by Harvard Medical School (138 publications), Stockholm University (112 publications), University of California, San Francisco (105 publications), and Chinese Academy of Sciences (104 publications; Table S4, Supplemental Digital Content 4). Consistently, in the top 10 institutions with the highest TLCS, the most institutions were from the United States, but there were no Chinese institutions (Table 2).

**Table 2:** Top 10 institutions with the highest TLCS.

Rank	Institution	Publications	TLCS	TGCS	Average TLCS
1	Harvard University	181	12,478	41,252	68.94
2	University of California, San Francisco	105	5344	15,909	50.90
3	University of Pennsylvania	73	4215	11,061	57.74
4	Stockholm University	112	3441	8635	30.72
5	University of Ancona	52	3135	8254	60.29
6	University of Turku	51	3019	7078	59.20
7	Dana-Farber Cancer Institute	41	2927	12,515	71.39
8	University of Gothenburg	34	2114	5755	62.18
9	LakePharma Inc.	2	1773	5637	886.50
10	Harvard Medical School	138	1712	6684	12.41

TGCS = total global citation score, TLCS = total local citation score.

**Figure 4.**
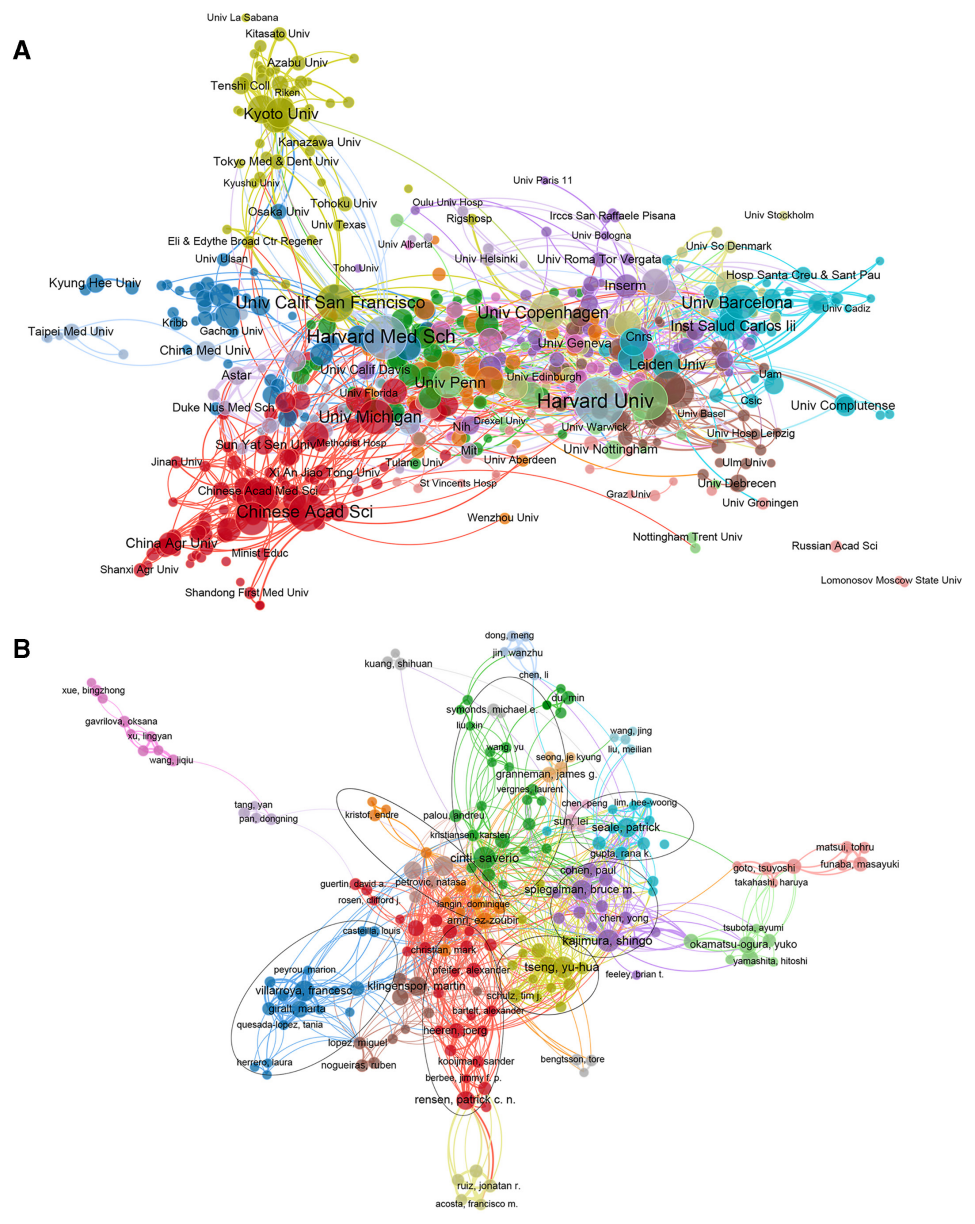
Active institutes and authors analysis. (A) Clustering of collaboration among institutes. (B) Clustering of collaboration among authors. In these maps, each institute or author is represented as a node, and each line represents a co-authorship relationship. The node size is proportional to the collaboration link strength, and the line thickness indicates the collaboration link strength. In addition, the node color reflects the cluster to which it belongs.

The co-authorship network of 609 institutions that published more than 5 papers was further analyzed, excluding those without connections, resulting in 606 institutions being considered. The result showed that Harvard Medical School had the most frequent collaborations with other institutions (total link strength = 433), followed by Harvard University (415), the University of Copenhagen (272), Chinese Academy of Sciences (254), and University of California, San Francisco (221; Fig. 4A). The collaborations among institutions can be divided into 15 clusters, with Harvard Medical School showing the most complex network of collaborations (Fig. 4A), indicating the crucial position of Harvard Medical School in thermogenic fat research.

### 3.4. Active authors in thermogenic fat research

A total of 21,584 authors contributed to publications on thermogenic fat. The author with the highest number of publications was Cinti Saverio from Marche Polytechnic University (83 articles; Table S5, Supplemental Digital Content 5), and Spiegelman was also among the top 10 authors with the highest TLCS (Table 3). This clearly demonstrates his significant contributions to the field of thermogenic fat (Table 3). In this study, a total of 194 authors who have published more than 10 papers were included in the coauthor analysis, excluding 35 authors without connections. Each node on the graph represents a different author. The size of the circle reflects the number of articles published by the researcher. Lines connecting the circles indicate collaborations between authors, with thicker lines indicating closer collaborations. The authors were divided into 7 major collaboration networks centered around Francesc Villarroya (total link strength = 132), Yu-Hua Tseng (130), Shingo Kajimura (127), Saverio Cinti (116), Patrick Resen (104), Ez-zoubir Amri (103), and Patrick Seale (82; Fig. 4B). These are the leading authors in collaborative research in this field.

**Table 3:** Top 10 authors with the highest TLCS.

Rank	Author	Publications	TLCS	TGCS	Institutions
1	Bruce M. Spiegelman	57	8597	26,116	Dana-Farber Cancer Institute
2	Shingo Kajimura	59	6669	16,137	Harvard University
3	Patrick Seale	47	5849	11,904	University of Pennsylvania
4	Saverio Cinti	83	4490	13,611	Marche Polytechnic University
5	Jan Nedergaard	74	3460	7917	Stockholm University
6	Sven Enerback	13	3038	6423	University of Gothenburg
7	Barbara Cannon	58	3021	7212	Stockholm University
8	Jun Wu	29	2888	8663	University of Michigan
9	Pirjo Nuutila	23	2875	6138	University of Turku
10	Kirsi A. Virtanen	26	2853	6176	University of Turku

TGCS = total global citation score, TLCS = total local citation score.

### 3.5. Core journals in thermogenic fat research

Source analysis of the included literature showed that thermogenic fat-related studies were published in 1009 journals, and the top 10 journals with the highest number of publications were listed in Table S6, Supplemental Digital Content 6, accounting for approximately 20.72% of the total literature. Among them, the journals with the highest number of publications were Molecular Metabolism (125 articles), Journal of Biological Chemistry (115), and Scientific Reports (114). Although journals such as Scientific Reports and Frontiers in Endocrinology have published more articles, these articles have been cited less frequently by peer articles. Table 4 lists the top 10 journals with the highest TLCS, accounting for 52.28% of the total TLCS, effectively reflecting their impact on the topic of thermogenic fat. Among them, Cell Metabolism has the highest TLCS, indicating its significant contribution and influence in this field. It is noteworthy that, although The New England Journal of Medicine has only published 3 articles on thermogenic fat research,^[18–20]^ it ranks tenth in TLCS (Table 4). This was mainly attributed to the article “Brief Report: Functional Brown Adipose Tissue in Healthy Adults,” which demonstrates that healthy adults possess BAT capable of expressing large amounts of UCP1 protein and containing more cytochrome C. The article also speculates that cold exposure activating BAT may be important for energy expenditure in humans.^[20]^

**Table 4:** Top 10 journals with the highest TLCS.

Rank	Journal	Counts	Impact factor (2022–2023)	TLCS	TGCS
1	Cell Metabolism	111	29	6938	19,881
2	Cell	31	64.5	3523	9621
3	Nature	34	64.8	3515	15,107
4	Nature Medicine	21	82.9	3235	7344
5	Journal of Biological Chemistry	115	4.8	2694	11,915
6	Diabetes	90	7.7	2048	8370
7	Journal of Clinical Investigation	32	15.9	1831	7302
8	Proceedings of the National Academy of Sciences of the United States of America	69	11.1	1718	6839
9	American Journal of Physiology-Endocrinology and Metabolism	99	5.1	1619	5081
10	The New England Journal of Medicine	3	158.5	1080	3470

TGCS = total global citation score, TLCS = total local citation score.

**Figure 5.**
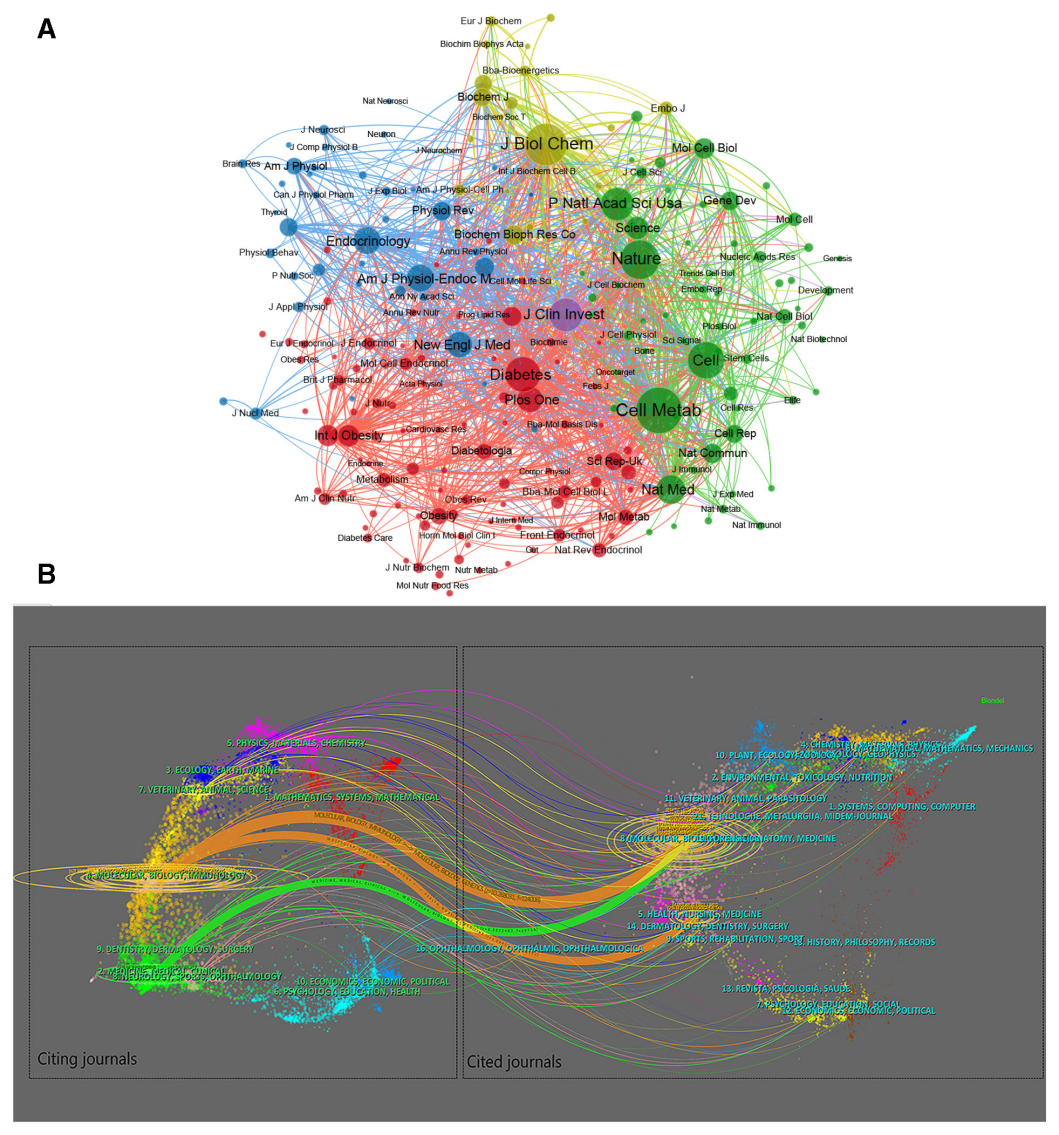
Core journals in the field of thermogenic adipocyte research. (A) Clustering of co-citation among journals. Each node represents a journal, and each line indicates a co-cited relationship. The node size is proportional to the co-cited link strength, and the node color reflects the cluster to which it belongs. (B) The dual-map overlay of articles citing thermogenic adipocyte research. The left and right sides are the citing and cited journals, respectively, and the line path represents the citation relationship.

A total of 218 journals with more than 200 citations were included in the co-citation analysis. Journals such as Cell Metabolism (total link strength = 1,058,299), Journal of Biological Chemistry (770,466), Nature (743,860), Cell (647,140), and Diabetes (634,123) had the most co-citations with other journals (Fig. 5A), indicating their significant roles in thermogenic fat research. The dual-map overlay of journals showed 3 main citation pathways. As the cited journals provide the knowledge base for citing journals, these citation pathways indicated that research on thermogenic fat mainly focuses on “Molecules, Biology, Immunology” and “Medicine, Medical Care, Clinical,” which were mainly based on research in “Molecules, Biology, Genetics” and “Health, Nursing, Medicine” (Fig. 5B).

### 3.6. Co-cited references in thermogenic fat research

Co-cited references represent the degree of relationship between references. The top 20 references with the highest TLCS are listed in Table 5. These references consisted of 15 original research papers and 5 review papers. Among these research papers, 8 focus on studying the conversion factors and activation pathways of inducible beige fat^[21–29]^; 6 investigate the transcriptional regulatory mechanisms of brown fat generation and energy expenditure in healthy adults,^[20,30–34]^ and only 1 is concerned with tracking fat generation during the development, expansion, and regeneration of WAT.^[35]^ Specifically, the reference with the highest TLCS was “Beige Adipocytes Are a Distinct Type of Thermogenic Fat Cell in Mouse and Human” by Wu et al in 2012 (TLCS = 1246). This was the first paper identifying beige adipocytes as a new type of thermogenic adipocyte in mice and humans, which was considered a breakthrough finding in the field of thermogenic fat.^[29]^ It is worth noting that, among the 15 original research papers, 4 explored brown or beige fat in adults, indicating that research on humans is more likely to gain recognition from peers and holds greater significance clinically.^[20,29–31]^ Besides, 5 papers investigated the role and function of PR domain containing 16 (PRDM16) in the development of brown fat and beige fat, suggesting the central role of PRDM16 in thermogenic fat regulation.^[23,25,28,32,33]^ Additionally, the 5 review papers primarily discussed the identity of beige fat, the functions of brown and beige fat, and their potential in treating metabolic diseases.^[24,36–39]^

**Table 5:** Top 20 publications with the highest TLCS.

Rank	First author	Journal	Year	TLCS	TGCS
1	Jun Wu	Cell	2012	1246	2406
2	Kirsi A. Virtanen	New England Journal of Medicine	2009	1027	2319
3	Matthew Harms	Nature Medicine	2013	850	1610
4	Patrick Seale	Nature	2008	840	1714
5	Masayuki Saito	Diabetes	2009	655	1430
6	Natasa Petrovic	Journal of Biological Chemistry	2010	569	1020
7	Pontus Bostroem	Nature	2012	527	3231
8	Patrick Seale	Journal of Clinical Investigation	2011	518	929
9	Patrick Seale	Cell Metabolism.	2007	493	872
10	Yu-Hua Tseng	Nature	2008	401	826
11	Shingo Kajimura	Cell Metabolism	2015	372	647
12	Haruya Ohno	Cell Metabolism	2012	367	582
13	Matthias Rosenwald	Nature Cell Biology	2013	334	575
14	Paul Cohen	Cell	2014	357	639
15	Aaron Martin Cypess	Nature Medicine	2013	346	520
16	Giorgio Barbatelli	American Journal of Physiology-Endocrinology and Metabolism	2010	334	551
17	Qiong A. Wang	Nature Medicine	2013	320	883
18	Alexander Bartelt	Nature Reviews Endocrinology	2014	315	773
19	Jun Wu	Genes & Development	2013	301	618
20	Labros Sidossis	Journal of Clinical Investigation	2015	301	475

TGCS = total global citation score, TLCS = total local citation score.

**Figure 6.**
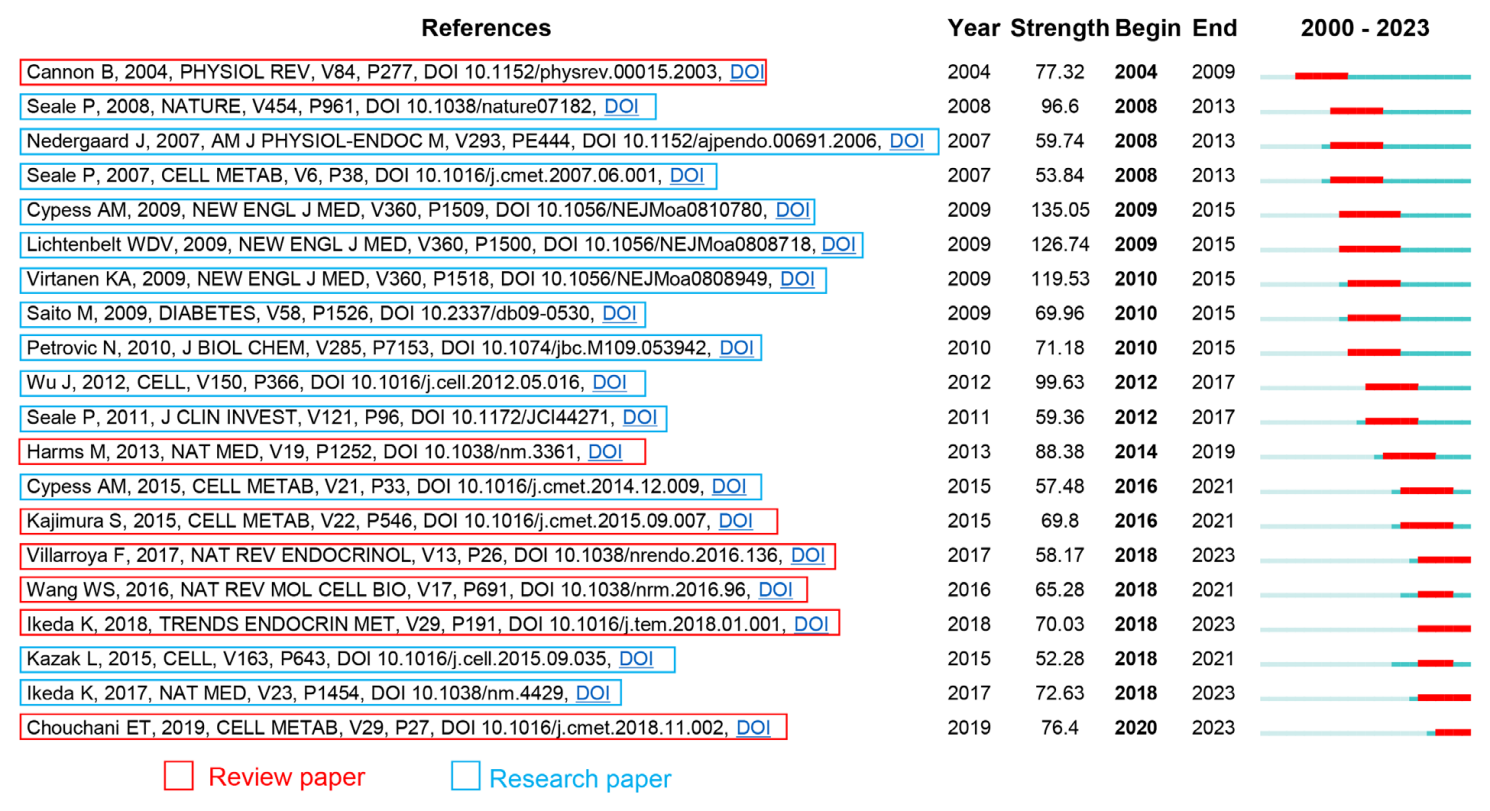
Top 20 references with the highest citation bursts of thermogenic adipocyte research.

Citation bursts refer to frequently cited references within a specific period, and the popularity of a particular topic within a certain time frame can be discovered through citation burst detection. Thus, in this study, citation burst detection was conducted, and the top 20 references with the strongest citation bursts were identified (Fig. 6). These references consisted of twelve original research papers and 8 review papers, underscoring the significance of both research and review papers in advancing science. Notably, 9 of these papers also ranked among the top 20 with the highest TLCS, indicating their substantial contributions to the field’s progress. Specifically, the review article published by Cannon et al in 2004 was recognized as the most systematic early review in the field of BAT.^[40]^ This paper comprehensively summarized the functions and physiological significance of BAT, laying a solid foundation for subsequent research and providing clear direction. From 2007 to 2009, Bruce M. Spiegelman and his colleagues discovered that BAT had a distinct cellular origin from WAT and elucidated the crucial role of PRDM16 in its origin and commitment.^[32]^ Concurrently, 5 different teams found that adults also possessed active BAT, a revelation that overturned the previous belief that BAT existed only in human infants and young children, significantly propelling the field’s development.^[20,31,41–43]^ Subsequently, from 2010 to 2013, researchers discovered that under the influence of cold stimuli and thermogenic inducers (such as irisin, Prdm16, Bmp8b, orexin, natriuretic peptides, and Sirt1), WAT had the potential to transform into other types of adipose tissue. This discovery provided a new perspective for understanding the treatment of obesity and its related metabolic disorders.^[24,26,28,29]^ After 2015, the research focus shifted to effectively increasing whole-body energy expenditure by activating the thermogenic mechanisms in BAT or promoting the development of beige fat cells.^[4,44–47]^ However, further research and validation are still needed for the clinical application of these compounds. Additionally, determining how various cell types in adipose tissue interact,^[48]^ as well as exploring the cues and mediators that promote the recruitment of beige fat cells,^[37]^ are also considered important areas of future research.

### 3.7. Keyword analysis

**Figure 7.**
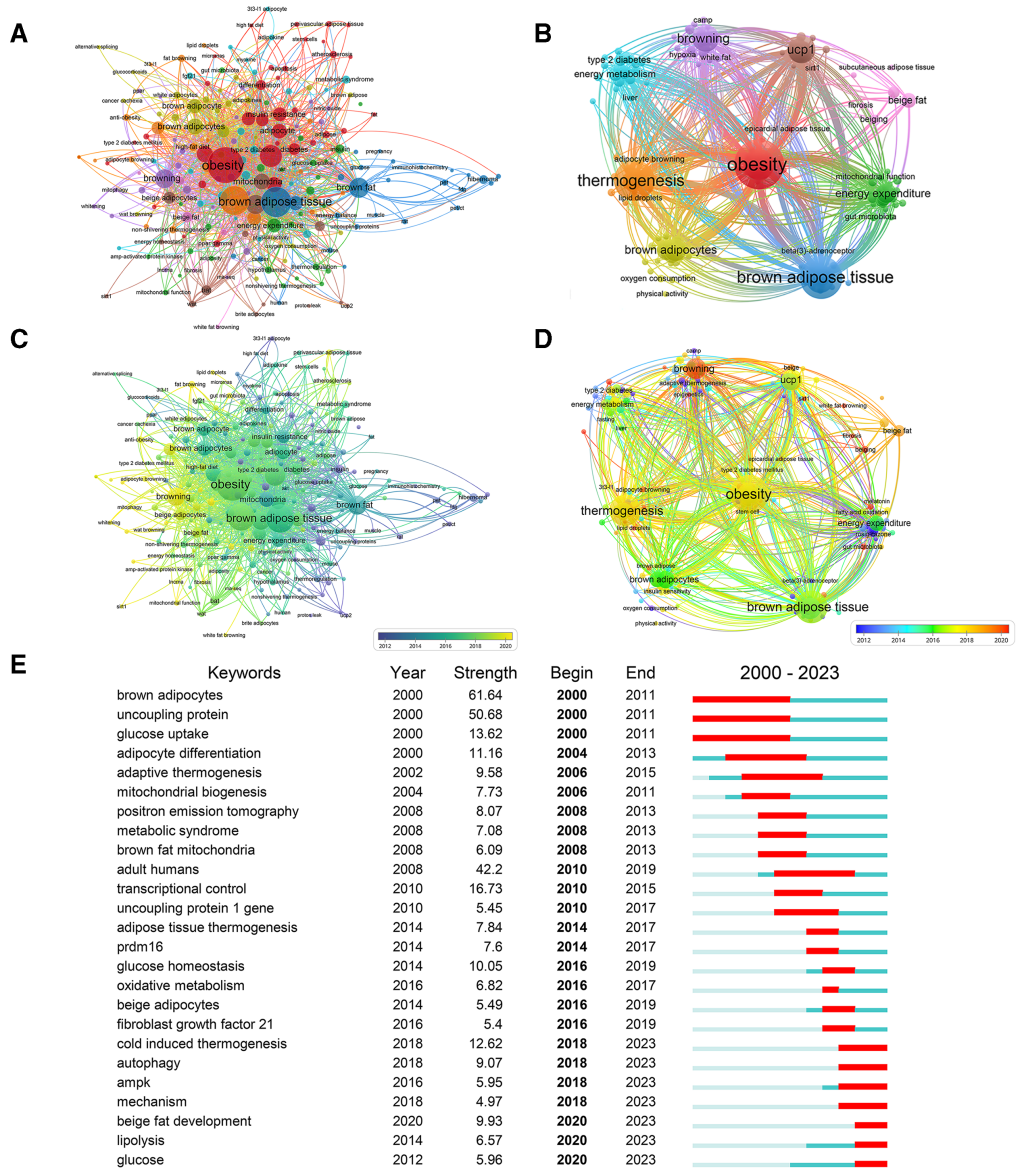
Analysis of keywords in publications. (A) Clustering of co-occurrence among keywords. Keywords with close relationships are assigned to 1 cluster with the same color. (B) Clustering of co-occurrences among high-frequency keywords. (C) Timeline visualization of co-occurrence among keywords. The nodes marked with purple color represent the keywords that appeared relatively earlier, whereas those with yellow color represent the current research focuses. (D) Timeline visualization of co-occurrence among high-frequency keywords. The nodes marked with purple color represent the keywords that appeared relatively earlier, whereas those with red color represent the current research focuses. (E) Top 25 keywords with the highest citation bursts.

Keyword co-occurrence networks could help us identify research hotspots and trends in a field. In this study, keyword analysis was performed, and the minimum number of occurrences of a keyword was set to 10, and a total of 205 keywords were extracted (Fig. 7A). Based on the cluster analysis, the research topic of hot fat production mainly focuses on the heat production of brown/beige fat and its mechanism, the regulatory factors of activating beige fat, and the transcriptional regulation, activation, and pharmacological targets and applications of hot fat production. The most frequently occurring keywords were obesity, BAT, thermogenesis, energy expenditure, brown adipocytes, browning, UCP1, beige adipocyte, and type 2 diabetes (Fig. 7B). To further explore hot topics and potential future topics, a time co-occurrence analysis of keywords was conducted (Fig. 7C and D). The analysis clearly showed that brown fat-related keywords appeared earlier, while the keyword “beige fat” appeared much later, which is highly consistent with the research trends in the field of thermogenic fat. Additionally, keyword burst detection was performed, and the top 25 keywords with the highest citations were shown in Figure 7E. The time interval is represented by a blue line, while the burst period is indicated by a red reflection line, which also shows the start and end years as well as the burst duration. To focus on terms indicating research trends in thermogenic fat, irrelevant keywords with little research significance were removed. As shown in Figure 7E, there were significant differences in the keywords with the strongest citation bursts across different time periods. In the early stages of this field, studies primarily focused on brown adipocyte differentiation and mitochondrial biogenesis. However, during the period from 2008 to 2014, with the application of positron emission tomography in assessing brown fat activity in adults and the discovery of active brown fat in adults, transcriptional regulation of brown fat mitochondrial biogenesis and thermogenesis, as well as their impact on metabolic syndrome, became research hotspots.^[20,41,43]^ Later, with the discovery of beige fat, exploring its function and biogenesis emerged as a new research focus.^[26]^ Interestingly, regardless of the stage, keywords such as “uncoupling protein,” “thermogenesis,” and “glucose” consistently appeared with high frequency, indicating that investigating the role and mechanism of thermogenic fat in glucose metabolism has always been a central focus of research.

## 4. Discussion

This study utilized bibliometric methods to systematically analyze research literature on thermogenic fat from 2000 to 2023. The findings revealed that this field of study has garnered extensive global attention, with a total of 5246 records retrieved from 1009 journals, involving 82 countries, 3918 institutions, and 21,584 authors. From the research results, a dramatic upward trend in the number of thermogenic fat-related publications was observed, especially after the discovery of active brown fat in adults in 2009.^[41]^ In terms of article output, the United States ranked first, followed by China, Germany, Japan, and Spain. Notably, except for China, the others were all developed nations, indicating the importance of socio-economic conditions for scientific research. It is worth noting that although China ranked second in terms of the number of papers published in this field, it ranked fifth in TLCS and tenth in average TLCS (Fig. 3B and C and Table 1), which indicates that the impact of papers published by Chinese scholars is limited. In the future, China needs to pay more attention to the quality rather than the quantity of papers. By contrast, besides the number of papers published, the United States ranked at the top both in TLCS and in cooperation with other countries, which demonstrates its leadership in this field.

The analysis of institutions and authors showed a similar pattern to that of countries. In the last decades, institutions in the United States, such as Harvard University and Harvard Medical School, have made outstanding contributions to this field and maintain strong collaborations with other institutions. Further analysis revealed that Saverio Cinti from Marche Polytechnic University has published the most articles, primarily focusing on the plasticity of adipose tissue, namely the interconversion between brown fat and white fat. By contrast, Francesc Villarroya from the University of Barcelona exhibited more complex collaborations with other authors, mainly concentrating on the molecular mechanisms of transcriptional control of the differentiation of thermogenic fat cells as well as how metabolic and endocrine functions are regulated in fat cells/tissues. Notably, Bruce M. Spiegelman, Shingo Kajimura, and Patrick Seale presented the top 3 most influential authors, as revealed by TLCS analysis. Given that Shingo Kajimura and Patrick Seale received training in Bruce M. Spiegelman’s lab, it is unquestionable that Bruce M. Spiegelman was a pioneer or an originator in this field.^[33,49]^

The field of thermogenic fat has not only attracted the attention of numerous researchers but also garnered significant interest from journal editors. According to our results, more than 1000 journals have published papers on this topic. Among them, the journal Molecular Metabolism published the most articles, while Cell Metabolism received the highest number of citations, and Cell had the highest average citation rate per paper. These results are of great significance for peers to choose appropriate journals when submitting manuscripts. Additionally, the top 3 most-cited journals were all top-tier publications, indicating their widespread recognition and significance to researchers in this field. It is worth noting that in this field, the articles that have garnered the highest citations were those that made significant and pioneering contributions to the identification, cellular origination, development, and functional understanding of brown and beige adipocytes.^[20,22,26,28,29,31,32]^ Consequently, these papers have attracted considerable attention and widespread acknowledgment from the scientific community. As such, they serve as invaluable references for emerging researchers in the field of thermogenic fat.

High-frequency keywords are usually used to identify research hotspots in a research field. The co-occurrence keyword analysis showed that obesity, brown fat tissue, thermogenesis, energy expenditure, browning, and beige fat were the major keywords in this field. However, it is worth noting that keywords such as brown fat, insulin resistance, diabetes, energy expenditure, differentiation, and PRDM16 appeared earlier, indicating that brown fat-related research is more advanced. The keywords related to beige fat appeared later, indicating that the research direction has changed from brown fat to beige fat. Currently, according to the citation burst analysis, exploring the mechanism of beige fat generation during cold-induced thermogenesis is a research hotspot.

## 5. Limitations

There were several limitations to this study. First, although our search strategy can cover the majority of studies related to thermogenic fat, it is inevitable that some literature, especially those published in journals not indexed by the Science Citation Index Expanded, will be missed. Second, due to limitation of the CiteSpace software, our data were limited to English literature, which may not be fully representative, potentially resulting in source bias. Additionally, the conclusions drawn by VOSviewer and CiteSpace were based on specific algorithms, which may have influenced the accuracy of the results to some extent. Lastly, as our study primarily relied on co-occurrence analysis of keywords and citations, it may not fully reveal potential future research trends.

## 6. Conclusion

To the best of our knowledge, this study is the first comprehensive bibliometric analysis of scientific literature concerning thermogenic fat research, utilizing quantitative and qualitative methodologies. Using advanced software tools, such as HistCite, CiteSpace, and VOSviewer, we generated visualization maps to illustrate annual publication trends, geographical distributions, institutional and authorial contributions, citation patterns, and keyword frequencies. Our findings reveal the significant contributions of the United States and its institutions in propelling thermogenic fat research forward. Notably, our investigation has uncovered a transition in research emphasis from brown to beige fat as the field has progressed. Nevertheless, research on both brown and beige fat remains primarily focused on topics pertaining to biogenesis, activation, maintenance, and involution. Although interest in thermogenic fat is escalating, there is a notable lack of clinical reports documenting its application in the treatment of obesity and associated metabolic disorders.

## Author contributions

**Conceptualization:** Mingsheng Jiang, Ziyi Song.

**Funding acquisition:** Ziyi Song.

**Data curation:** Haiyan Xie, Lan Chen.

**Visualization:** Haiyan Xie, Lan Chen.

**Investigation:** Boyuan Long.

**Project administration:** Mingsheng Jiang, Ziyi Song.

**Resources:** Ziyi Song.

**Supervision:** Mingsheng Jiang, Ziyi Song.

**Writing—original draft:** Haiyan Xie, Lan Chen, Boyuan Long, Xudong Song.

**Writing—review & editing:** Mingsheng Jiang, Ziyi Song.












