# Supplementary material for: Past, present, and future of thermogenic fat research: A bibliometric analysis from 2000 to 2023
Source: Medicine (Baltimore). 2026 Jun 12;105(24):e49210. doi: 10.1097/MD.0000000000049210 (PMC13268563; doi:10.1097/MD.0000000000049210)
Supplement: Supplementary file 1 [file medi-105-e49210-s001.docx]

**Supplementary Table S1.** Excluded publication

| Type | Title |
| --- | --- |
| Retracted publication | RETRACTED: Bi-directional regulation of brown fat adipogenesis by the insulin receptor (Retracted article. See vol. 291, pg. 27434, 2016) |
|  | RETRACTED: Protein Phosphatase PP5 Controls Bone Mass and the Negative Effects of Rosiglitazone on Bone through Reciprocal Regulation of PPARγ (Peroxisome Proliferator-activated Receptor γ) and RUNX2 (Runt-related Transcription Factor 2) (Retracted article. See vol. 293, pg. 8314, 2018) |
|  | RETRACTED: miR-127 aggravates myocardial failure by promoting the TGF-1/Smad3 signaling (Retracted article. See vol. 20, pg. 2500, 2019) |
