# Supplementary material for: Past, present, and future of thermogenic fat research: A bibliometric analysis from 2000 to 2023
Source: Medicine (Baltimore). 2026 Jun 12;105(24):e49210. doi: 10.1097/MD.0000000000049210 (PMC13268563; doi:10.1097/MD.0000000000049210)
Supplement: Supplementary file 2 [file medi-105-e49210-s002.docx]

**Supplementary Table S2.** Publication types concerning thermogenic fat

| Document Type | Number | TLCS | TGCS |
| --- | --- | --- | --- |
| Article | 4,413 | 43,913 | 206,444 |
| Review | 833 | 9721 | 51,748 |
