# Supplementary material for: Past, present, and future of thermogenic fat research: A bibliometric analysis from 2000 to 2023
Source: Medicine (Baltimore). 2026 Jun 12;105(24):e49210. doi: 10.1097/MD.0000000000049210 (PMC13268563; doi:10.1097/MD.0000000000049210)
Supplement: Supplementary file 3 [file medi-105-e49210-s003.docx]

**Supplementary Table S3.** The top 10 productive countries concerning thermogenic fat

| Rank | Country | Publications | TLCS | TGCS | Average TGCS |
| --- | --- | --- | --- | --- | --- |
| 1 | USA | 1,998 | 32,243 | 146,131 | 73.14 |
| 2 | China | 994 | 3,992 | 25,163 | 25.31 |
| 3 | Germany | 460 | 4,689 | 24,528 | 53.32 |
| 4 | Japan | 429 | 3,540 | 16,551 | 38.58 |
| 5 | Spain | 352 | 3,147 | 16,778 | 47.66 |
| 6 | South Korea | 311 | 1,028 | 7,398 | 23.79 |
| 7 | UK | 298 | 3,295 | 17,855 | 59.92 |
| 8 | France | 267 | 3,256 | 16,625 | 62.27 |
| 9 | Italy | 258 | 5,118 | 20,303 | 78.69 |
| 10 | Sweden | 220 | 7,649 | 20,778 | 94.45 |
