# Supplementary material for: Past, present, and future of thermogenic fat research: A bibliometric analysis from 2000 to 2023
Source: Medicine (Baltimore). 2026 Jun 12;105(24):e49210. doi: 10.1097/MD.0000000000049210 (PMC13268563; doi:10.1097/MD.0000000000049210)
Supplement: Supplementary file 4 [file medi-105-e49210-s004.docx]

**Supplementary Table S4.** The top 10 productive institutions concerning thermogenic fat

| Rank | Institution | Publications | TLCS | TGCS | Average TGCS |
| --- | --- | --- | --- | --- | --- |
| 1 | Harvard University | 181 | 12,478 | 41,252 | 227.91 |
| 2 | Harvard Medical School | 138 | 1,712 | 6,684 | 48.43 |
| 3 | Stockholm University | 112 | 3,441 | 8,635 | 77.10 |
| 4 | University of California, San Francisco | 105 | 5,344 | 15,909 | 151.51 |
| 5 | Chinese Academy of Sciences | 104 | 681 | 3,478 | 33.44 |
| 6 | University of Copenhagen | 92 | 1,510 | 6,959 | 75.64 |
| 7 | University of Michigan | 90 | 943 | 5,600 | 62.22 |
| 8 | Technical University of Munich | 84 | 1,045 | 3,984 | 47.43 |
| 9 | University of Barcelona | 84 | 1,629 | 5,870 | 69.88 |
| 10 | Shanghai Jiao Tong University | 78 | 279 | 1,817 | 23.29 |
