# Supplementary material for: Past, present, and future of thermogenic fat research: A bibliometric analysis from 2000 to 2023
Source: Medicine (Baltimore). 2026 Jun 12;105(24):e49210. doi: 10.1097/MD.0000000000049210 (PMC13268563; doi:10.1097/MD.0000000000049210)
Supplement: Supplementary file 5 [file medi-105-e49210-s005.docx]

**Supplementary Table S5.** The top 10 active authors concerning thermogenic fat

| Rank | Name | Publications | TLCS | TGCS | Institutions |
| --- | --- | --- | --- | --- | --- |
| 1 | Saverio Cinti | 83 | 4,490 | 13,611 | Marche Polytechnic University |
| 2 | Jan Nedergaard | 74 | 3,460 | 7,917 | Stockholm University |
| 3 | Francesc Villarroya | 65 | 1,588 | 5,205 | Universitat de Barcelona |
| 4 | Yu-Hua Tseng | 63 | 2,560 | 6,313 | Harvard Medical School |
| 5 | Shingo Kajimura | 59 | 6,669 | 16,137 | Harvard University |
| 6 | Barbara Cannon | 58 | 3,021 | 7,212 | Stockholm University |
| 7 | Bruce M. Spiegelman | 57 | 8,597 | 26,116 | Dana-Farber Cancer Institute |
| 8 | M Klingenspor | 49 | 868 | 2,359 | Technische Universität München |
| 9 | Marta Giralt | 47 | 1,340 | 4,318 | Universitat de Barcelona |
| 10 | Patrick Seale | 47 | 5,849 | 11,904 | University of Pennsylvania |
