# Supplementary material for: Past, present, and future of thermogenic fat research: A bibliometric analysis from 2000 to 2023
Source: Medicine (Baltimore). 2026 Jun 12;105(24):e49210. doi: 10.1097/MD.0000000000049210 (PMC13268563; doi:10.1097/MD.0000000000049210)
Supplement: Supplementary file 6 [file medi-105-e49210-s006.docx]

**Supplementary Table S6.** The top 10 active journal concerning thermogenic fat

| Rank | Journal | Publications | Impact factor (2022-2023) | TLCS | TGCS | H index |
| --- | --- | --- | --- | --- | --- | --- |
| 1 | Molecular Metabolism | 125 | 8.1 | 775 | 2,935 | 43 |
| 2 | Journal of Biological Chemistry | 115 | 4.8 | 2,694 | 11,915 | 477 |
| 3 | Scientific Reports | 114 | 4.6 | 0 | 2,531 | 149 |
| 4 | Plos One | 113 | 3.7 | 0 | 4,733 | 268 |
| 5 | Cell Metabolism | 111 | 29 | 6,938 | 19,881 | 219 |
| 6 | International Journal of Molecular Sciences | 111 | 5.6 | 2 | 1,722 | 114 |
| 7 | Frontiers in Endocrinology | 106 | 5.2 | 0 | 2,899 | 51 |
| 8 | Endocrinology | 102 | 4.8 | 877 | 5,393 | 241 |
| 9 | American Journal of Physiology-Endocrinology and Metabolism | 99 | 5.1 | 1,619 | 5,081 | 182 |
| 10 | Nature Communications | 91 | 16.6 | 0 | 4,668 | 248 |
